# Supplementary material for: Horizontal transfer of chromosomal DNA mediated by an integrative and conjugative element generates frequent localized recombination in Novosphingobium aromaticivorans
Source: J Bacteriol. 2026 Aug 7;208(9):e00175-26. doi: 10.1128/jb.00175-26 (PMC13584701; doi:10.1128/jb.00175-26)
Supplement: Supplementary file 1 [file jb.00175-26-s0001.pdf]

Supplementary Information

Horizontal transfer of chromosomal DNA mediated by an integrative and conjugative element generates frequent localized recombination in *Novosphingobium aromaticivorans*

Marco N. Allemann[1, †], Leah H. Hochanadel[1,†], Delyana P. Vasileva[1,2], and Joshua K. Michener[1,2]

[1]Biosciences Division and [2]Center for Bioenergy Innovation, Oak Ridge National Laboratory, Oak Ridge, TN. [†]: These authors contributed equally to this work

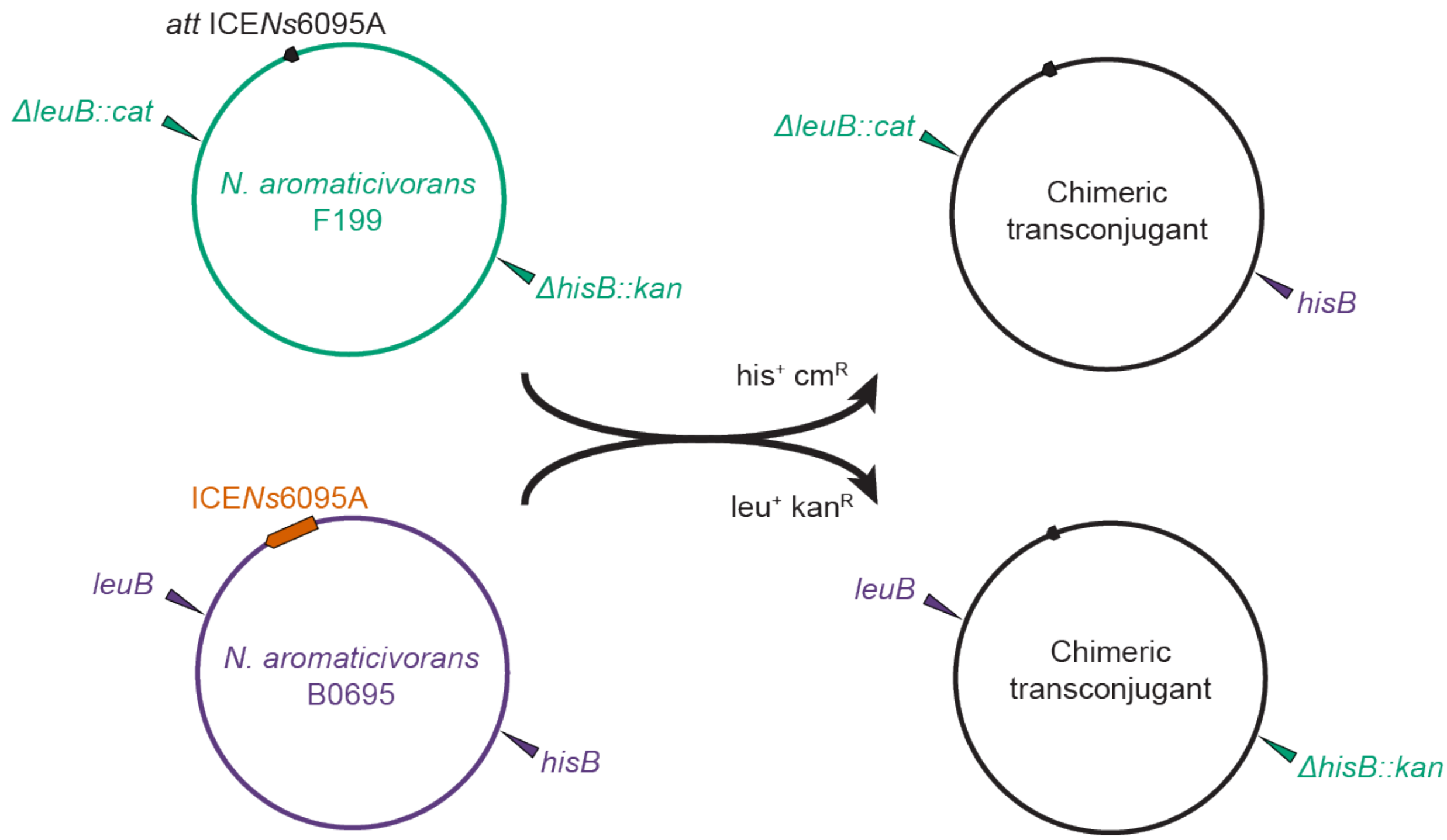

Figure S1: Recombination between *N. aromaticivorans* F199 (green) and B0695 (purple). Selection/counterselection markers are indicated with colored arrows. The location of ICE*Ns*6095A and its associated *att* site are shown in red and black, respectively.

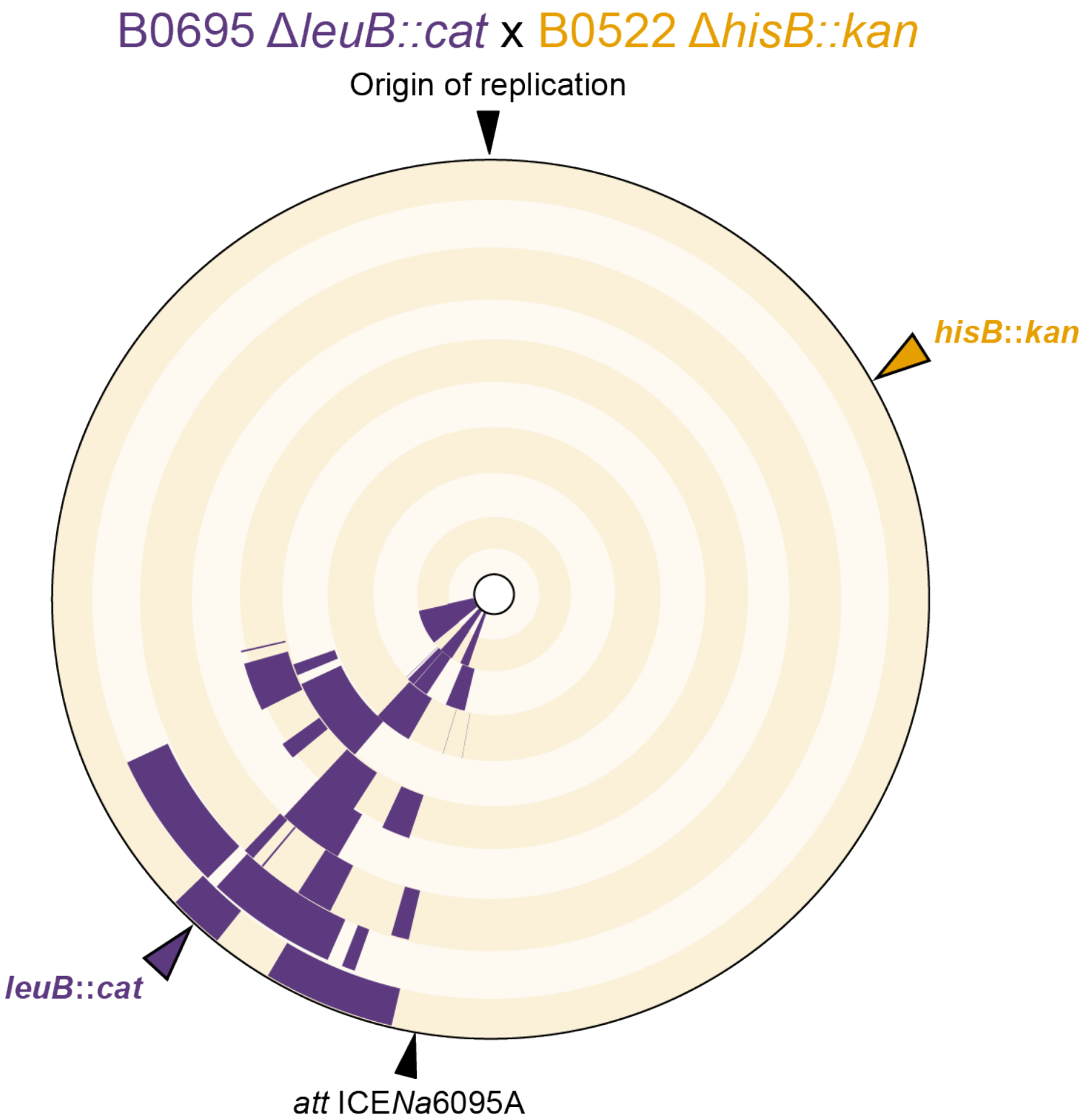

Figure S2: Chimeric genomes of kan^{R} cm^{R} progeny from crossing B0695 *leuB::cat* (purple) with B0522 *hisB::kan* (orange). Each concentric circle represents the genome of a single progeny strain. The majority of the genome was contributed by B0522. Regions highlighted in purple are derived from B0695. The location of the selection markers are highlighted with colored arrows. Black arrows indicate the origin of replication and the *att* site for ICE*Na*6095A.

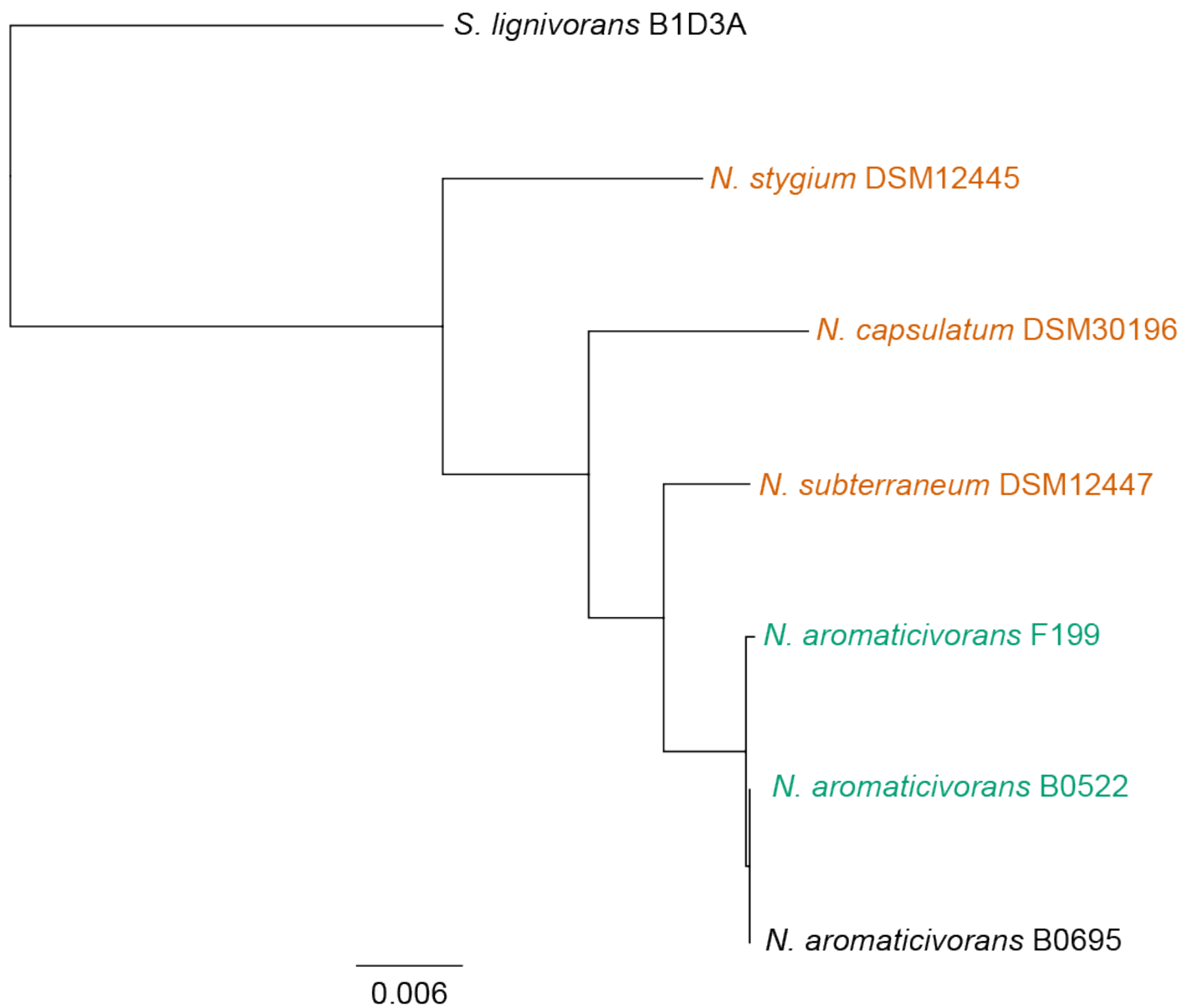

Figure S3: 16S rRNA phylogeny of *Novosphingobium* strains used in this work. *Sphingobium lignivorans* B1D3A was used as an outgroup. DNA was successfully recombined from the donor, B0695, into B0522 and F199 (green) but not into *N. subterraneum*, *N. capsulatum*, or *N. stygium* (red). *N. subterraneum* has 83.1% average nucleotide identity with *N. aromaticivorans* F199. *N. capsulatum* has 78.7% average nucleotide identity with *N. aromaticivorans* F199. The phylogenetic tree was constructed using the Tamura-Nei genetic model and the neighbor-joining method.

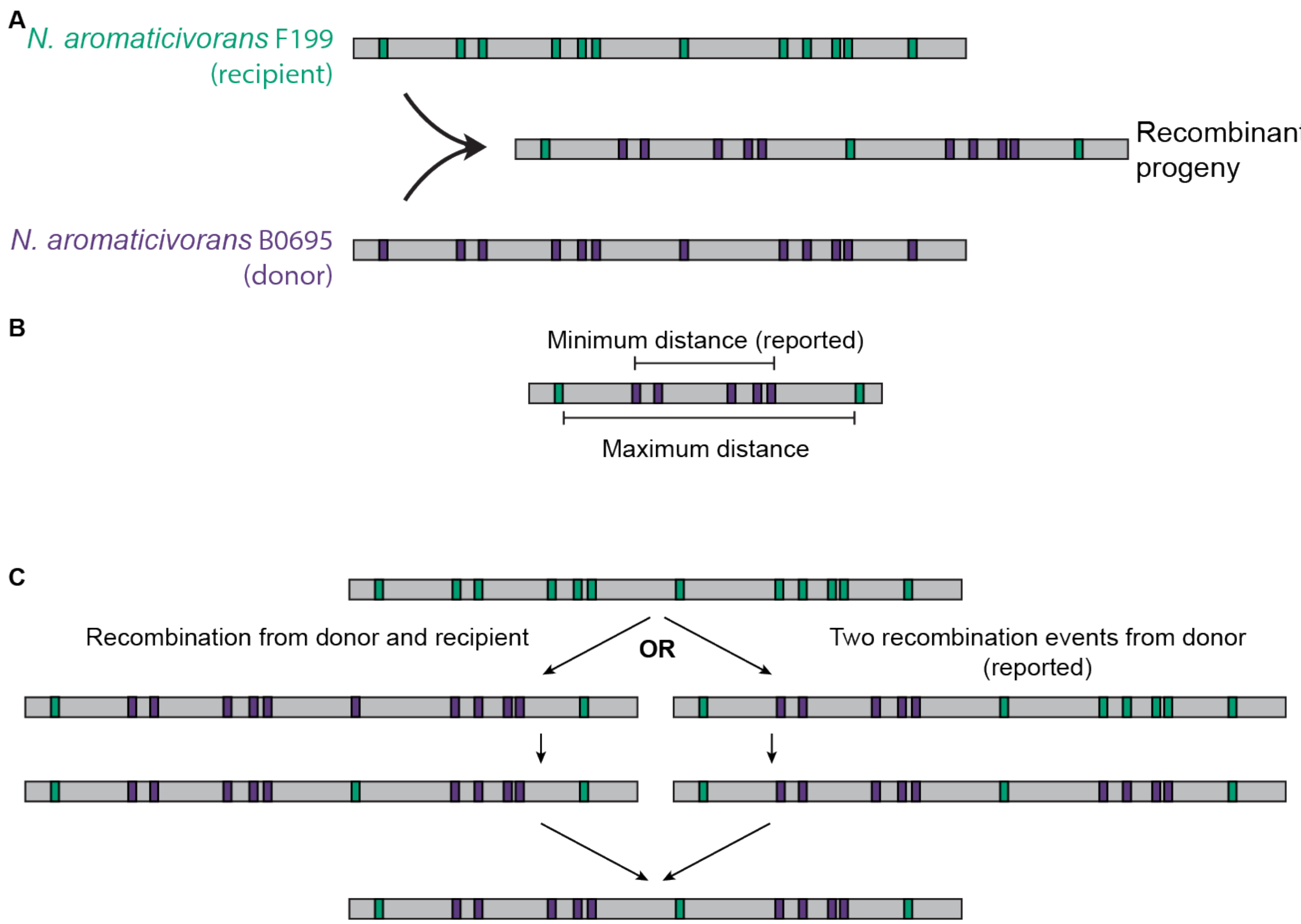

Figure S4: Analysis of chimeric genomes. (A) Schematic genomes of F199 and B0695 showing conserved DNA (grey) and strain-specific genetic variants (green and purple). Recombination generates chimeric progeny (middle) with mixtures of parental variants. (B) Due to the conserved DNA that is identical in both parents, the exact length of physically recombined DNA cannot be determined. We report the minimum distance between introgressed variants, though the true fragment length could be longer. Conserved (grey) segments are, on average, 143 bp in length. (C) Multiple introgressed fragments could be due to sequential recombination of shorter fragments (right) or recombination of a large donor fragment followed by reversion of a smaller internal fragment (left). Since these alternatives cannot be precisely differentiated based on resequencing data, we report this scenario as two separate donor-derived fragments.

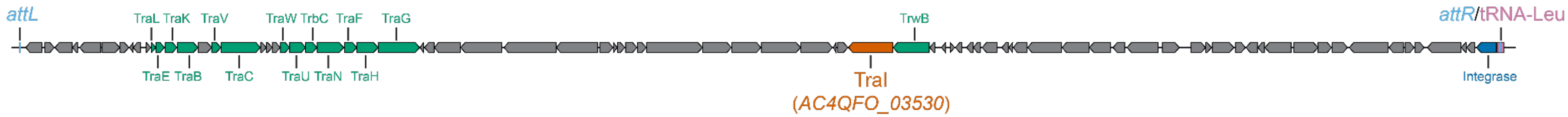

Figure S5: Genetic structure of ICENa0695A. The ICE is predicted to integrate between two *att* sites (light blue, ccagaagaggactcg), one located in a leucine tRNA gene (pink). The associated putative site-specific integrase is shown in dark blue. The predicted relaxase *traI* (*AC4QFO_03530*) is shown in red. Genes encoding the type IV secretion system are shown in green.

Table S1: Summary of crosses

Parents	Progeny phenotype	Selection	Progeny
B0695 x<br>F199 Δ*hisB*::*kan* Δ*leuB*::*cat*	kan^R leu^+<br>cm^S his^-	+kanamycin<br>+histidine	Figure 1; SRA SAMN61125391, SAMN61125392, SAMN61125393, SAMN61125394, SAMN61125395, SAMN61125396, SAMN61125397, SAMN61125398, SAMN61125399, SAMN61125400, SAMN61125401
B0695 x<br>F199 Δ*hisB*::*kan* Δ*leuB*::*cat*	cm^R his^+<br>kan^S leu^-	+chloramphenicol<br>+leucine	Figure 1; SRA SAMN61125402, SAMN61125403, SAMN61125404, SAMN61125405
JMN150 (F199 Δ*hisB*::*kan*) x<br>JMN127 (F199 Δ*leuB*::*cat*)	cm^R kan^R	+chloramphenicol<br>+kanamycin	Figure 3
JMN146 (F199C Δ*hisB*::*kan*) x<br>JMN 149 (F199C Δ*leuB*::*cat*)	cm^R kan^R	+chloramphenicol<br>+kanamycin	Figure 3
B0695 x<br>JMN 154 (F199 Δ*hisB*::*kan* Δ*leuB*::*cat*)	kan^R leu^+<br>cm^S his^-	+kanamycin<br>+histidine	Figure 4
JMN219 (B0695 Δ*traI::cat*) x<br>JMN154 (F199 Δ*hisB*::*kan* Δ*leuB*::*cat*)	kan^R leu^+<br>cm^S his^-	+kanamycin<br>+histidine	Figure 4
